# Preparation and Properties of Cyano-Functionalized Graphitic Nanoplatelets for High-Performance Acrylonitrile Butadiene Styrene Resin

**DOI:** 10.3390/polym16202859

**Published:** 2024-10-10

**Authors:** Seo-Jeong Yoon, Se-Jung Lee, Jae-Hoon Baek, Tae-Hee Kim, In-Yup Jeon

**Affiliations:** 1 Department of Chemical Engineering, Nanoscale Environmental Sciences and Technology Institute, Wonkwang University, 460 Iksandae-ro, Iksan 54538, Republic of Korea; dbstjwjd1210@wku.ac.kr (S.-J.Y.); lse1222@wku.ac.kr (S.-J.L.); 2 School of Energy and Chemical Engineering, Center for Dimension-Controllable Covalent Organic Frameworks, Ulsan National Institute of Science and Technology (UNIST), UNIST-gil 50, Ulsan 44919, Republic of Korea; baekjhid@unist.ac.kr

**Keywords:** graphitic nanoplatelets, cyano-functionalization, mechanochemical reaction, acrylonitrile butadiene styrene, compatibility

## Abstract

Cyano-functionalized graphitic nanoplatelets (CyGNs) are synthesized by means of a mechanochemical reaction between graphite and acrylonitrile. The resulting CyGNs exhibit excellent mechanical properties and are highly dispersible in various solvents (i.e., THF). Due to their chemical compatibility (specifically, cyano functional groups), the CyGNs serve effectively as a reinforcing filler for acrylonitrile butadiene styrene (ABS) resin. Consequently, compared to pure ABS, CyGN&ABS-X demonstrates improved mechanical properties and better thermal stability. Notably, the CyGN&ABS-1 specimen exhibits significant enhancements in the tensile strength (26 ± 1 MPa), Young’s modulus (992 ± 71 MPa), and tensile toughness (22 ± 3 MPa), representing increases of approximately 130.6%, 19.2%, and 59.6%, respectively, over pure ABS. This underscores the ability of a mechanochemical reaction to directly modify the functional groups of graphitic nanoplatelets (GnPs) as fillers, facilitating their strong compatibility with a variety of polymers, including copolymers.

## 1. Introduction

Carbon-based nanofillers in polymer nanocomposites have been widely studied as part of the effort to create multifunctional materials with customized properties, such as enhanced mechanical, thermal, and electrical performance capabilities [1,2]. Such polymer nanocomposites are used in various fields, including the automotive, aerospace, and chemical industries, and in electronic enclosures [3]. The properties of polymer nanocomposites rely mainly on the mixing quality, degree of stabilization, and the presence of a uniform dispersion [4], especially for nanosized dispersions. Therefore, a good dispersion is a crucial factor related to mechanical strengthening between a filler and a polymer matrix. This is a significant challenge, as the natural tendency of nanofillers (i.e., agglomerates) hinders an effective dispersion. Therefore, covalent modification of nanofillers is essential and effective for improving their dispersion properties [5].

The properties of the filler, including its shape, size, dispersion, content, and the filler–polymer interactions, are crucial in determining the overall mechanical and physical characteristics of polymer nanocomposites. Diverse forms of fillers, such as one-dimensional (1D), two-dimensional (2D), and three-dimensional (3D) types, have been integrated into different styles of polymer matrices to study their effects on the resulting thermal, mechanical, and rheological properties [6,7,8,9]. Among the different geometric shapes, nanocomposites exhibit the most significant property enhancements up to a certain loading level, influenced by the aspect ratio of the filler. The improvement in the mechanical properties can be attained at lower concentrations.

Graphene, a single layer of carbon atoms arranged in a hexagonal lattice with *sp^2^* bonds, shows outstanding electrical and mechanical properties owing to its unique electronic configurations and minimal thickness [10,11]. The drive to utilize uniformly dispersed nanocomposites stems from the need for materials with improved mechanical properties essential for diverse applications [12]. Graphene has a 2D geometry that results in a high surface-to-volume ratio and offers very high aspect ratios. Its exceptional tensile strength and Young’s modulus make it ideal as a reinforcing material in polymer nanocomposites, enhancing their mechanical properties significantly [13,14,15].

The present authors pioneered an efficient mechanochemical process capable of producing graphitic nanoplatelets (GnPs) in large quantities without the need for additional chemical reactions [16,17,18,19,20]. In relation to this, cyano-functionalized graphitic nanoplatelets (CyGNs) were cyano-functionalized. Cyano-functionalized graphitic nanoplatelets (CyGNs) were created with graphite and acrylonitrile to use a new additive for polymers. The CyGNs showed distinctive properties (i.e., cyano-functionalization, a high surface area, excellent dispersibility, among others). They especially showed excellent chemical affinity with acrylonitrile butadiene styrene (ABS) resin given their cyano-functionalization. Accordingly, different CyGN&ABS-X (CyGN loading (X) = 1, 2, or 5 wt.%) specimens were created through a solution method. It was found that these materials exhibited significantly improved mechanical properties and good thermal stability in comparison with pure ABS resin.

## 2. Materials and Methods

### 2.1. Materials

Graphite, obtained from Alfa Aesar (Natural, −100 mesh, 99.9995% metals basis), was used as received. Acrylonitrile (≥99%, containing 35–45 ppm monomethyl ether hydroquinone as inhibitor) was obtained from Aldrich Chemical, Inc. and was also used as received. Acrylonitrile butadiene styrene resin (ABS, HI-121-H-NP) was acquired from LG Chem Ltd. and was used as received. Unless otherwise stated, other solvents were acquired from Aldrich Chemical, Inc. and were used without further purification.

### 2.2. Preparation of Cyano-Functionalized Graphitic Nanoplatelets (CyGN)

Graphite (5.0 g), acrylonitrile (5 mL) without an inhibitor, and stainless-steel balls (500 g, diameter 5 mm) were put into a stainless-steel container. The container was tightly locked and evacuated to remove air using a vacuum pump. Subsequently, the sealed container was placed in a planetary mill and rotated at 500 rpm. After 48 h, the resulting material was collected and treated with 1 M aqueous HCl and acetone to eliminate completely metallic impurities and unreacted acrylonitrile, respectively. Lastly, the product was lyophilized for 48 h, yielding 8.6 g of a dark black powder (containing approximately 3.6 g of acrylonitrile).

### 2.3. Preparation of CyGN&ABS-X Film

The CyGNs were dispersed into THF using a tip sonicator for 1 h. The pure ABS resin was soluble in THF at 60 °C. The CyGN&THF solution was slowly poured into the ABS&THF solution, and the mixed solution was stirred at 60 °C. After 24 h, this solution was slowly poured into a glass Petri dish, after which the THF was gradually removed initially at 40 °C for 24 h and then at 60 °C for 24 h. Lastly, the resultant film was thoroughly dried under reduced pressure (0.5 mmHg) at 60 °C for 12 h to obtain the CyGN&ABS-X film (where X represents the CyGN content). For tensile testing, the prepared films were cut into dumbbell-shaped specimens measuring 79 mm × 4.3 mm × 0.3 mm (L × W × T).

### 2.4. Instrumentation

The morphologies were examined by FE-SEM (Nanonova 230, FEI, Hillsboro, OR, USA) and high-resolution transmission electron microscopy (HR-TEM, JEM-2100F, JEOL, Tokyo, Japan). Fourier transform infrared (FT-IR) spectroscopy was utilized with a Perkin-Elmer Spectrum 100 instrument with KBr disks. A surface area analysis was carried out by N_2_ adsorption–desorption isotherms using the Brunauer–Emmett–Teller (BET) method on a Micromeritics ASAP 2504N (Micromeritics, Norcross, GA, USA). X-ray diffraction (XRD) patterns were measured on a Rigaku D/MAZX 2500V/PC with Cu-Kα radiation (35 kV, 20 mA, λ = 1.5418 Å). An elemental analysis (EA) was conducted using a Thermo Scientific Flash 2000 instrument. The X-ray photoelectron spectra (XPS) were obtained using a Thermo Fisher K-alpha X-ray photoelectron spectrometer. A thermogravimetric analysis (TGA) was conducted using a TA Q200 (TA Instruments) device under air and N_2_ atmospheres at a heating rate of 10 °C min^−1^. A dynamic mechanical analysis (DMA) was carried out using DMA 8000 instrument (PerkinElmer) in tension mode, with the temperature ranging from 30 °C to 120 °C at a heating rate of 2 °C min^−1^ and a frequency of 1 Hz. The tensile properties of the films were determined at room temperature using a universal testing machine (DR101, DRTECH Co., Seong-nam, Republic of Korea) at a strain rate of 20 mm min^−1^.

## 3. Results and Discussion

**Figure 1:**
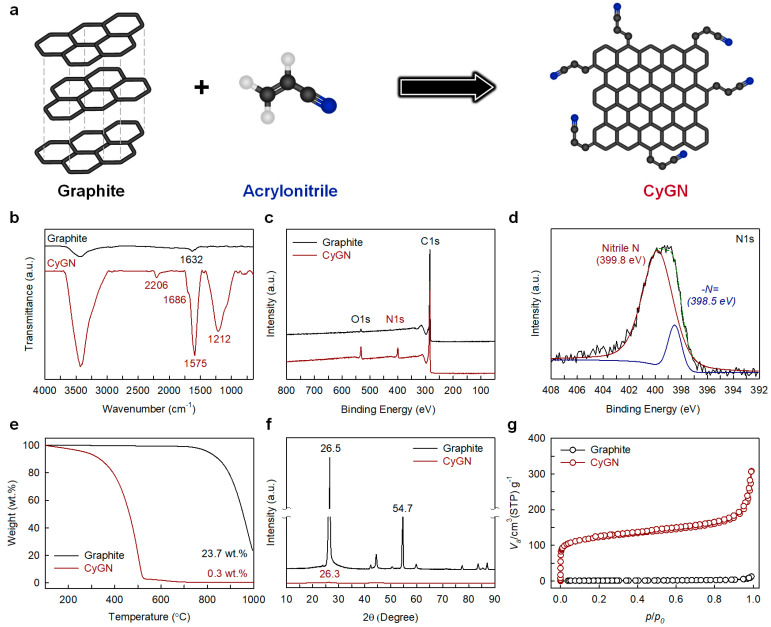
(**a**) Schematic diagram of the mechanochemical reaction when using graphite and acrylonitrile. Characteristic comparison between the pristine graphite and CyGN: (**b**) FT−IR spectra, (**c**) XPS survey spectra, (**d**) high−resolution XPS spectra of N1s for the CyGN, (**e**) TGA curve in air, (**f**) XRD spectra, and (**g**) N_2_ adsorption−desorption isotherms.

First, a mechanochemical reaction between pristine graphite and acrylonitrile could directly produce cyano-functionalized graphitic nanoplatelets (CyGNs) without requiring additional reactions (Figure 1a). The resulting products with metal impurities and residual acrylonitrile were removed with 1 M aq. HCl and acetone, respectively, ensuring the purity of the CyGN specimens.

FT-IR analysis, as depicted in Figure 1b, is valuable because it allows for the determination of the composition and structure of functional groups through an analysis of the position, width, and intensity of infrared light absorption. The pristine graphite exhibited peaks at 1632 cm^−1^ related to the vibration mode of the adsorbed H_2_O molecules [21], whereas the CyGNs showed characteristic peaks, C≡N (2206 cm^−1^) [22,23], indicating that acrylonitrile was bonded chemically to them. In addition, the CyGNs showed various peaks at 1686 cm^−1^ (C=O), 1575 cm^−1^ (aromatic C=C), and 1212 cm^−1^ (C–O), corresponding to the oxygen functional groups that arose during its preparation [16,17,24].

X-ray photoelectron spectroscopy (XPS) is another valuable analysis approach by which to assess chemical structures and bonding properties. The pristine graphite exhibited prominent C1s and minor O1s peaks, indicative of the physical adsorption of oxygen on its surface [25,26]. However, the CyGN specimen displayed a sharp C1s peak and more obvious O1s and N1s peaks related to its functional groups (Figure 1c). Observing the high-resolution XPS spectra, the C1s peak could be separated into four peaks, C=C (284.7 eV), C–O (285.8 eV), nitrile (287.7 eV), and C=O (290.1 eV) (Figure S1a), while the O1s peak could be divided into two peaks, C=O (531.2 eV) and C–O (532.7 eV) (Figure S1b). Additionally, the N1s peak could be separated into two peaks: nitrile N (399.8 eV) and –N= (398.5 eV) (Figure 1d) [27,28]. Subsequently, FT-IR and XPS demonstrated that the CyGNs are functionalized by cyano (or nitrile, C≡N) units through a mechanochemical reaction with the pristine graphite and acrylonitrile.

To evaluate the quantitative functionality of the CyGN, a thermogravimetric analysis (TGA) was conducted. In an air condition, the pristine graphite was stabilized above 800 °C, whereas the CyGNs exhibited significant weight loss (Figure 1e) due to their very fine particle size (less than 1 µm) and numerous functional groups (e.g., cyano and oxygen functional groups). In this case, the residue levels of the pristine graphite and CyGNs amounted to 23.7% and 0.3%, respectively, at 1000 °C. In a N_2_ condition, the pristine graphite remained stable, whereas the CyGNs displayed continuous weight loss up to 1000 °C (Figure S2), indicating that numerous cyano functional groups exist on the CyGNs. The residue levels of the pristine graphite and CyGNs amounted to 99.1% and 10.5%, respectively, at 1000 °C.

In Figure 1f, the XRD spectra of the pristine graphite showed 26.5° (002) and 54.7° (004) peaks that correspond to the C-axis direction vertical to the graphite planes. In contrast, the CyGNs showed only a 26.3° (002) peak, moved by about 0.2° with the peak intensity reduced to 0.07% in comparison with pristine graphite. The shifting and significant decline of the (002) peak’s intensity, along with the absence of a (004) peak, imply that thick graphite layers were peeled into thin layers owing to the wedge effect of the cyano functional groups in the CyGNs.

To use N_2_ adsorption–desorption isotherms with the Brunauer–Emmett–Teller (BET) method, the specific surface areas (SSAs) of the pristine graphite (2.8 m^2^ g^−1^) and CyGNs (453.4 m^2^ g^−1^) were determined (Table S2). The SSAs of the CyGNs were increased by approximately 162 times in comparison with those of the pristine graphite due to exfoliation into few-layer graphitic sheets caused by the wedge effect of the cyano functional groups at the edges of the CyGNs. Moreover, the CyGN specimen exhibited type-I isotherms and type-H3 hysteresis (P/P° = 0.5~1.0) (Figure 1g), signifying its microporous and sheet-like structure, or slit-type pores [29,30].

**Figure 2:**
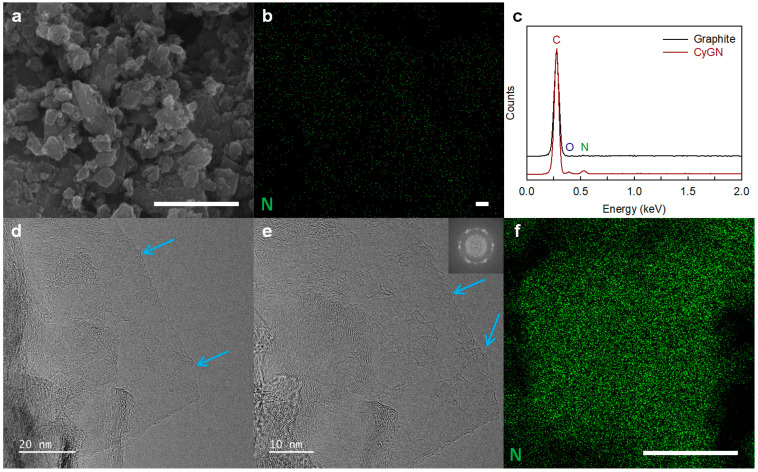
(**a**) FE-SEM image, (**b**) corresponding nitrogen mappings, and (**c**) energy dispersive X-ray (EDX) spectra of the CyGNs. (**d**,**e**) HR-TEM images (inset: FFT pattern) and (**f**) corresponding nitrogen (N) mappings of the CyGNs. Scale bars represent 1 µm.

The morphology of the pristine graphite and CyGNs was analyzed by means of FE-SEM. The pristine graphite presented a heavy layer shape with very large pieces (Figure S3), while the CyGNs displayed a spherical structure with particle sizes smaller than 1 µm (Figure 2a and Figure S4). The sharp structure of the pristine graphite was completely fragmented by the kinetic energy of the metal balls, resulting in the generation of activated carbon species (e.g., radicals and ions) from the broken carbon bonds, which subsequently reacted with acrylonitrile to produce the CyGNs. The SEM EDX spectrum of the CyGNs expressed peaks corresponding to C, O, and a distinctive N (Figure 2c), with corresponding atomic percentages of 82.87, 6.70, and 10.43 (Table S1). Elemental mapping images of the CyGN (Figure 2b and Figure S6) specimens confirmed the presence of cyano-functionalization.

According to high-resolution transmission electron microscopy (HR-TEM), the CyGNs have a few-layered, highly ordered graphitic structure with a honeycomb lattice in the basal region (Figure 2d,e and Figure S5). Additionally, the fast Fourier transform (FFT; inset of Figure 1e) of the CyGNs clearly display a six-fold pattern. The CyGNs maintain a well-ordered graphitic basal plane and some edge distortion (Figure 2d,e, sky blue arrow) despite the cyano-functionalization via a mechanochemical reaction involving high kinetic energy. Elemental mapping images of the CyGNs (Figure 2f and Figure S6) confirmed the presence of nitrogen (N) associated with the cyano functional groups.

The outstanding dispersion of CyGNs as a filler in various solvents (Figure S7) indicates their strong potential in polymer nanocomposites. Accordingly, CyGN&ABS-X specimens were conveniently fabricated through a THF-based solution method. Based on the characteristic analysis of the CyGNs, these results imply that their exceptional properties, including cyano-functionalization, excellent dispersibility, and a high surface area, contribute to enhancing the properties of ABS resin.

**Figure 3:**
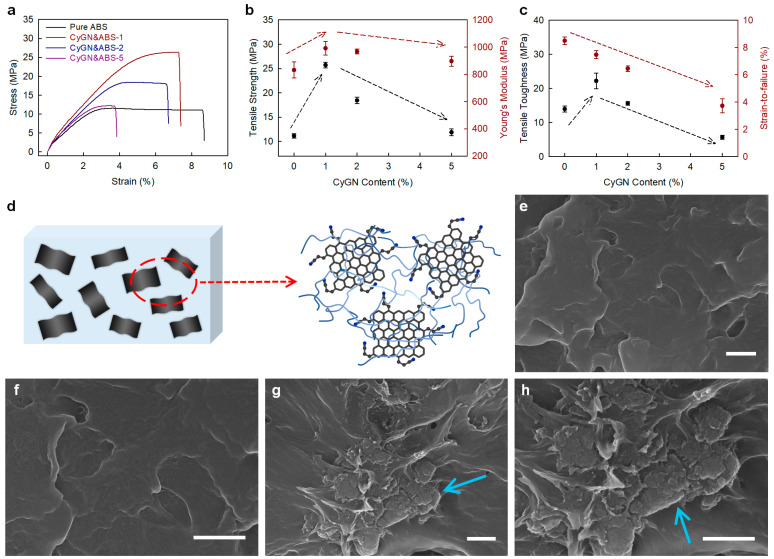
Mechanical properties: (**a**) stress–strain curves, (**b**) tensile strength and Young’s modulus, and (**c**) tensile toughness and elongation. (**d**) Scheme of the interaction between the CyGNs and ABS chain. FE-SEM images of the broken surfaces after the tensile test: (**e**,**f**) pure ABS and (**g**,**h**) CyGN&ABS-1. Scale bars represent 5 µm.

Tensile tests of pure ABS and CyGN&ABS-X (CyGN loading (X): 1, 2, or 5 wt.%) were executed with dumbbell-shaped specimens (Figure S8). As shown in Figure 3a and Figure S9, the stress–strain curves in pure ABS and CyGN&ABS-X cases revealed that the loading of the CyGNs had a decisive influence on the mechanical properties (i.e., tensile strength, Young’s modulus, tensile toughness, and elongation). In a comparison with pure ABS (11.1 ± 0.4, 832 ± 65, and 13.9 ± 1.3 MPa), the tensile strength, Young’s modulus, and tensile toughness of the CyGN&ABS-X specimens were found to have increased substantially (Figure 3b,c and Table 1). Among these specimens, the CyGN&ABS-1 specimen exhibited the highest tensile strength (25.7 ± 0.6 MPa), Young’s modulus (992 ± 71 MPa), and tensile toughness (22.2 ± 3.2 MPa), presenting corresponding increases of approximately 131%, 19.2%, and 59.7% compared to pure ABS. However, the elongation of the CyGN&ABS-X specimen was reduced consistently with an increase in the amount of CyGN loading.

The significant improvements in tensile strength and Young’s modulus of the CyGN&ABS-X specimen result from the even distribution of the CyGNs within the ABS matrix, promoting strong interfacial adhesion and enhanced π–π interactions between the ABS chains and CyGNs. This effective stress transfer from the pure ABS to the CyGNs is facilitated by the exceptional intrinsic properties of the CyGNs, such as their high specific surface area, microporous structure, and layered morphology. However, beyond a CyGN content of 1 wt.%, the tensile strength, Young’s modulus, and tensile toughness of the CyGN&ABS-X specimens decline due to the CyGNs’ aggregation within the ABS matrix. This suggests that the optimal degree of CyGN incorporation into the ABS matrix occurs at loadings around 1 wt.%

Furthermore, the elongation of the CyGN&ABS-X specimens decreased with an increase in the CyGN loading level (Figure 3c and Figure S9), an outcome attributed to the enhanced interaction between the polymers and the multidimensional filler, which restricts movement of the polymer chain. The CyGNs, being rigid and exhibiting minimal elongation, are uniformly dispersed within the pure ABS, forming physical cross-linking sites that impede the mobility of the polymer chain (Figure 3d). Consequently, while the tensile strength and Young’s modulus of the CyGN&ABS-X specimens were improved significantly, the elongation was reduced. Nonetheless, the CyGN&ABS-5 specimen exhibited higher tensile strength and a higher Young’s modulus compared to those of pure ABS.

XRD patterns of both pure ABS and CyGN&ABS-X (Figure S10) specimens exhibited broad peaks, indicating the amorphous nature of the CyGN&ABS-X specimens, which incorporate ABS resin. XRD peak intensities in the CyGN&ABS-X were higher than those of pure ABS. Specifically, the CyGN&ABS-1 showed the highest intensity, with the intensity changes correlating with the mechanical property trends observed in the pure ABS and CyGN&ABS-X. This confirms the significant role of the CyGNs as a filler material in the ABS matrix.

After the tensile tests, the fractured surfaces between pure ABS and CyGN&ABS-1 specimens showed a clear distinction. The pure ABS displayed an even and soft surface (Figure 3e,f), but the CyGN&ABS-1 specimen showed a rough and gnarled surface (Figure 3g,h). Specifically, the CyGN&ABS-1 specimen showed a shredded, fractured surface (Figure 3g,h, sky blue arrow), signifying that this specimen could tolerate the applied stress but becomes sheared through an efficacious load transfer between the CyGNs and the ABS chain and the physical cross-linking parts of the CyGNs.

**Figure 4:**
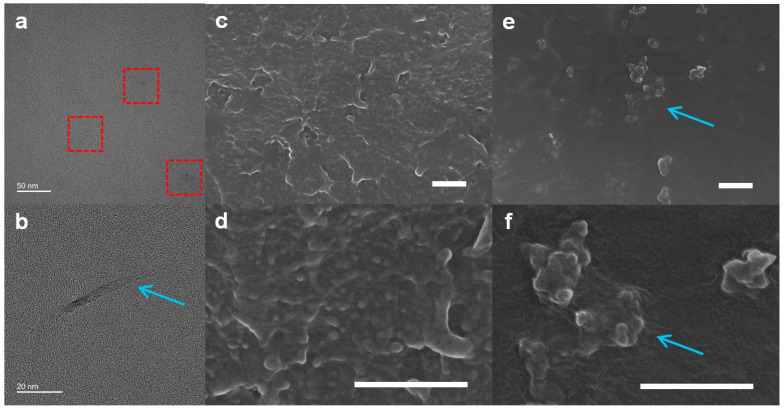
(**a**,**b**) HR-TEM images of the CyGN&ABS-1 specimen. FE-SEM images of the freeze-fractured surfaces of CyGN&ABS-1 are shown (**c**,**d**) before and (**e**,**f**) after a heat treatment. The scale bars represent 5 µm.

The dispersion and organization of the CyGN into the ABS resin are very important factors to consider when interpreting the increased performance of the CyGN&ABS-X specimens. Accordingly, the morphologies of microtomed sections of the films were investigated by HR-TEM (Figure 4a,b). In the TEM images, the CyGNs were uniformly dispersed (Figure 4a, red box) without agglomeration, and the boundary between the CyGNs and ABS resin was ambiguous (Figure 4b, sky blue arrow). Therefore, the unique characteristics (i.e., cyano-functionalization and nanoscopic size) of the CyGNs allowed for an increase in their interactions with the ABS resin and induced an increase in interfacial areas.

An interrelation between the mechanical properties and the dispersion of the CyGNs in the ABS chain was confirmed. The CyGN&ABS-1 specimen was frozen in liquid nitrogen and then broken (Figure 4c,d), with these pieces then treated by heating in N_2_ at 100 °C (Figure 4e,f). The CyGN specimens were capped tightly with the ABS resin and scattered uniformly in the ABS matrix (Figure 4e,f, sky blue arrow). It was found that the cyano functional groups of the CyGNs remarkably enhance the chemical compatibility and dispersibility and reinforce the performance of the CyGN&ABS-X specimen.

**Figure 5:**
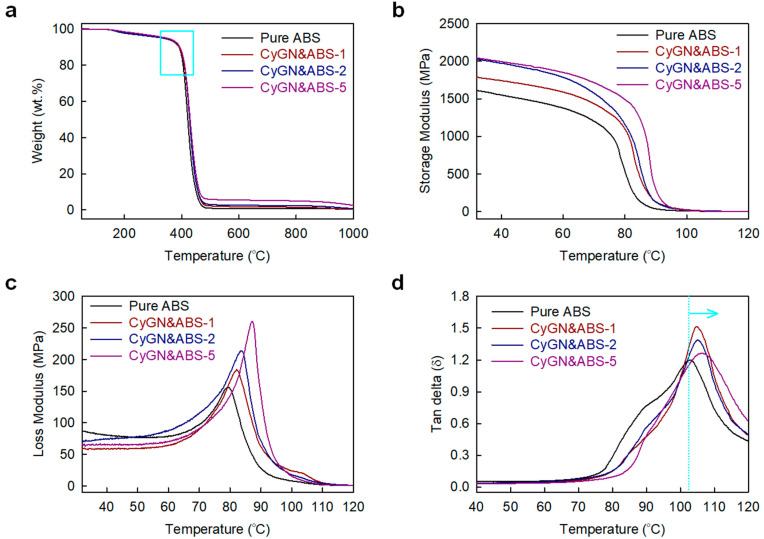
Thermal properties of pure ABS and CyGN&ABS-X. (**a**) TGA curves in air; (**b**–**d**) DMA curves: (**b**) storage modulus, (**c**) loss modulus, and (**d**) tan delta.

An examination of the TGA curves (Figure 5a and Figure S11) indicated that the CyGNs significantly enhanced the thermal stability of the ABS resin. The temperature at which 10% weight loss occurred (T_d10%_) for pure ABS was 390.2 °C, whereas in the CyGN&ABS-X specimen, the corresponding temperatures were 390.5 °C, 393.2 °C, and 393.1 °C, respectively (Table S3). The slightly increased thermal stability of the CyGN&ABS-X specimen can be ascribed to the even dispersion of the CyGNs with a very high aspect ratio in the ABS resin, as the formation of small thermally degraded gaseous molecules is hindered.

The effects of the CyGNs on ABS nanocomposites regarding the dynamic mechanical characteristics were assessed through a dynamic mechanical analysis (DMA). Figure 5b–d depict the storage modulus, loss modulus, and tan delta (δ) of both the materials tested here across different temperatures. Figure 5b clearly shows that all CyGN&ABS-X specimens exhibit a significantly higher storage modulus compared to pure ABS, indicating a considerable increase in the tensile modulus (stiffness) due to the presence of the CyGNs. Additionally, the CyGN&ABS-X specimen demonstrated a higher loss modulus and greater loss factor than pure ABS (Figure 5c,d), suggesting greater energy dissipation and damping during cyclic deformation, stemming from the large interfacial area and increased interfacial friction sliding between the CyGNs and the ABS chains. These enhanced dynamic mechanical properties result from an enhanced network within the polymer matrix, facilitating efficacious load transfers from the rubber domain to the CyGNs. Moreover, cyano functional groups improve the molecular dispersion of the graphite flakes, which might decrease the mobility of the polymer chains.

In a comparison with pure ABS, the CyGNs induced a slight increment in the glass transition temperature (T_g_) in the CyGN&ABS-X specimen. Moreover, an increase in the CyGN content increases the T_g_ in the CyGN&ABS-X specimens (Figure 5d and Table S3). This suggests that the CyGNs contributed to nucleation and cross-linking effects. Furthermore, this finding aligns well with the enhancement of the mechanical properties in ABS resin by the CyGNs given its cyano functional groups.

## 4. Conclusions

To summarize, we synthesized, for the first time, cyano-functionalized graphitic nanoplatelets (CyGNs) from graphite and acrylonitrile by means of a mechanochemical reaction, which is a direct and straightforward process requiring no additional reactions. Characterization of the synthesized CyGNs using various analytical techniques revealed excellent dispersion in various solvents and exceptional chemical compatibility with the ABS matrix due to cyano-functionalization. Consequently, the CyGN&ABS-X specimens were easily prepared via a solution process and displayed exceptional mechanical properties and good thermal stability. In particular, the CyGN&ABS-1 specimen demonstrated the best tensile strength (25.7 ± 0.6 MPa), Young’s modulus (992 ± 71 MPa), and tensile toughness (22.2 ± 3.2 MPa), achieving increases of approximately 131%, 19.2%, and 59.7% compared to pure ABS, respectively. Furthermore, we reaffirmed that a mechanochemical reaction can effectively handle the functional groups at the edges of graphitic nanoplatelets (GnPs), highlighting their significant potential as novel reinforcing fillers for polymers.

## Figures and Tables

**Table 1:** Mechanical properties of pure ABS and CyGN&ABS-X.

	Tensile Strength (MPa)	Young’s Modulus (MPa)	Tensile Toughness (MPa)	Elongation (%)
Pure ABS	11.1 ± 0.4	832 ± 65	13.9 ± 1.3	8.5 ± 0.2
CyGN&ABS-1	25.7 ± 0.6	992 ± 71	22.2 ± 3.2	7.5 ± 0.4
CyGN&ABS-2	18.4 ± 1.0	968 ± 21	15.6 ± 0.7	6.5 ± 0.3
CyGN&ABS-5	11.9 ± 0.9	897 ± 52	5.7 ± 0.8	3.7 ± 0.7

## Data Availability

Data are contained within the article or Supplementary Materials.

## Supplementary Materials

The following supporting information can be downloaded at: https://www.mdpi.com/article/10.3390/polym16202859/s1, Figure S1: High-resolution XPS spectra of the CyGN: (a) C1s; (b) O1s; Figure S2. TGA curves of the pristine graphite and CyGN in N_2_; Figure S3. FE-SEM image of the pristine graphite. Scale bar is 1 µm; Figure S4. FE-SEM images of the CyGN. Scale bars are 1 µm; Figure S5. HR-TEM images of the CyGN; Figure S6. (a) FE-SEM and (b) HR-TEM images of the CyGN. Corresponding element mappings: (c and d) Carbon; (e and f) Oxygen. Scale bars are 1 µm; Figure S7. Photographs of the CyGN dispersed solutions in various solvents on bench top in a normal laboratory condition: (a) after 30 s; (b) after 1 week; Figure S8. Photographs images of dumbbell-shaped test specimen: (a) pure ABS; (b) CyGN&ABS-1; Figure S9. The stress-strain curves of the pure ABS and CyGN&ABS-X; Figure S10. XRD patterns of the pure ABS and CyGN&ABS-X; Figure S11. The magnified TGA curves of sky blue box in Figure 5a; Table S1: TGA, EA, EDS, and XPS data of the pristine graphite and CyGN; Table S2. BET surface area, pore volume, and pore size of the pristine graphite and CyGN; Table S3. Thermal properties of the pure ABS and CyGN&ABS-X.
