# Supplementary material for: Preparation and Properties of Cyano-Functionalized Graphitic Nanoplatelets for High-Performance Acrylonitrile Butadiene Styrene Resin
Source: Polymers (Basel). 2024 Oct 10;16(20):2859. doi: 10.3390/polym16202859 (PMC11511425; doi:10.3390/polym16202859)
Supplement: Supplementary file 1 [file polymers-16-02859-s001.zip › polymers-3232146-supplementary.pdf]

Supporting Information

Preparation and Properties of Cyano-Functionalized Graphitic Nanoplatelets for High-Performance Acrylonitrile Butadiene Styrene Resin

Seo-Jeong Yoon [1], Se-Jung Lee [1,2], Jae-Hoon Baek [2], Tae-Hee Kim [1,*] and In-Yup Jeon [1,*]

[1] Department of Chemical Engineering, Nanoscale Environmental Sciences and Technology Institute, Wonkwang University, 460 Iksandae-ro, Iksan, Jeonbuk 54538, Republic of Korea

[2] School of Energy and Chemical Engineering, Center for Dimension-Controllable Covalent Organic Frameworks, Ulsan National Institute of Science and Technology (UNIST), UNIST-gil 50, Ulsan 44919, Republic of Korea

* Correspondence: taehee928@wku.ac.kr (T.-H.K.); iyjeon79@wku.ac.kr (I.-Y.J.)

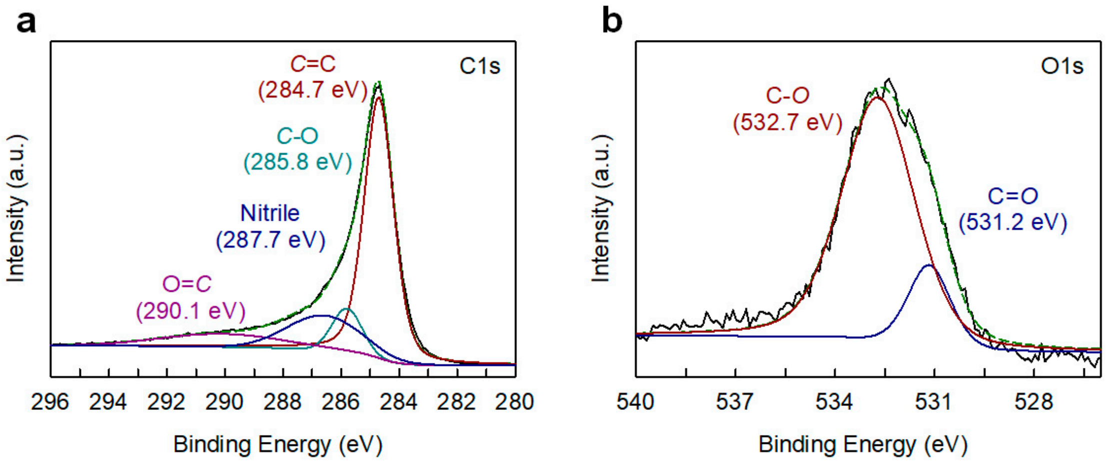

Figure S1. High-resolution XPS spectra of the CyGN: (a) C1s; (b) O1s.

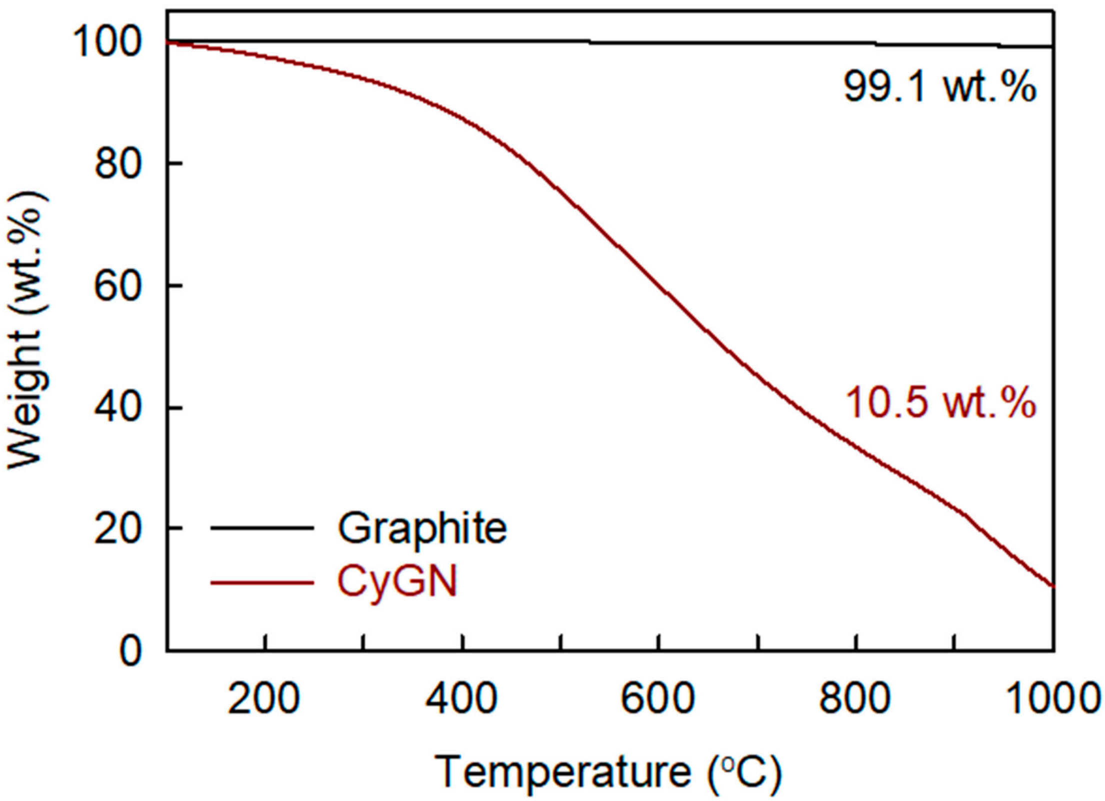

Figure S2. TGA curves of the pristine graphite and CyGN in N_2.

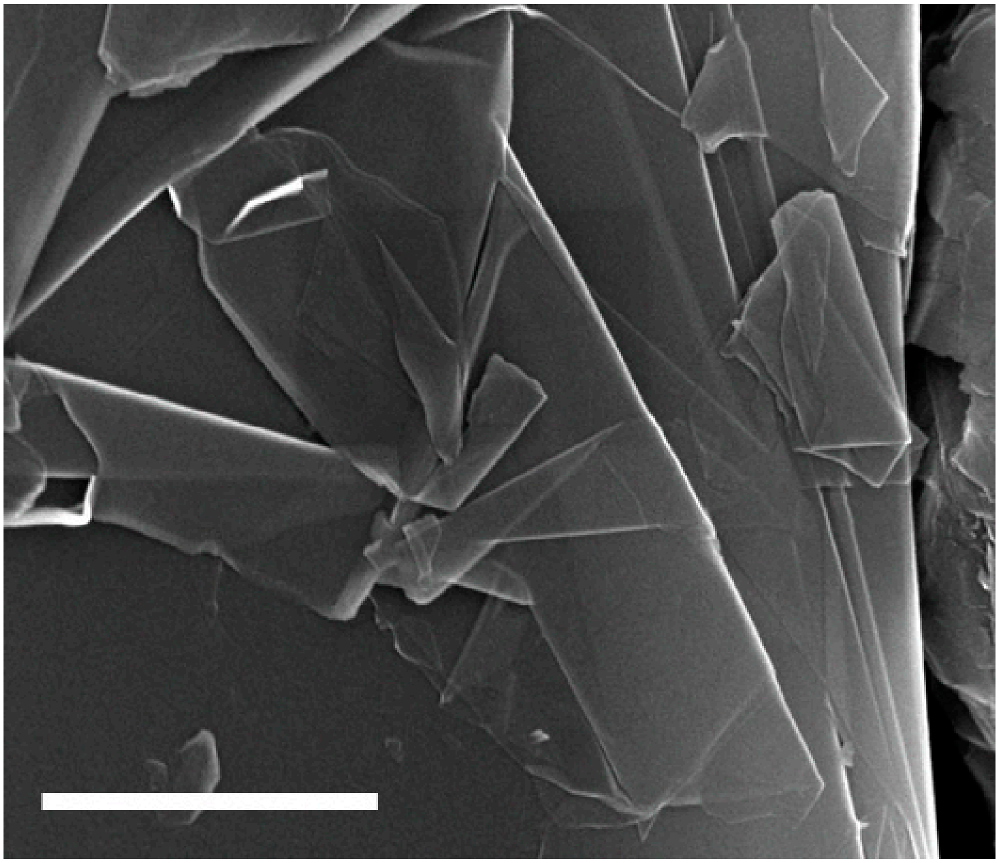

Figure S3. FE-SEM image of the pristine graphite. Scale bar is 1 μm.

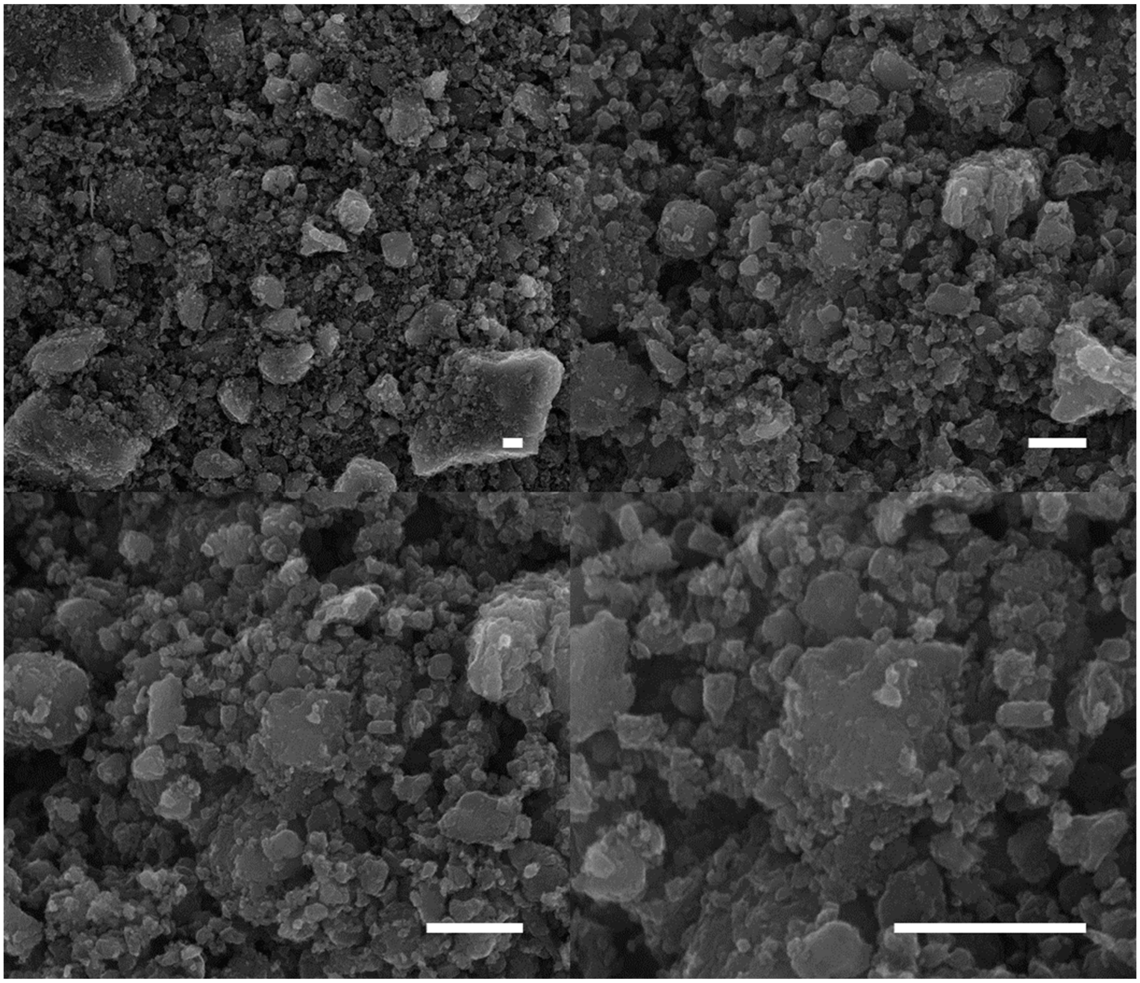

Figure S4. FE-SEM images of the CyGN. Scale bars are 1 μm.

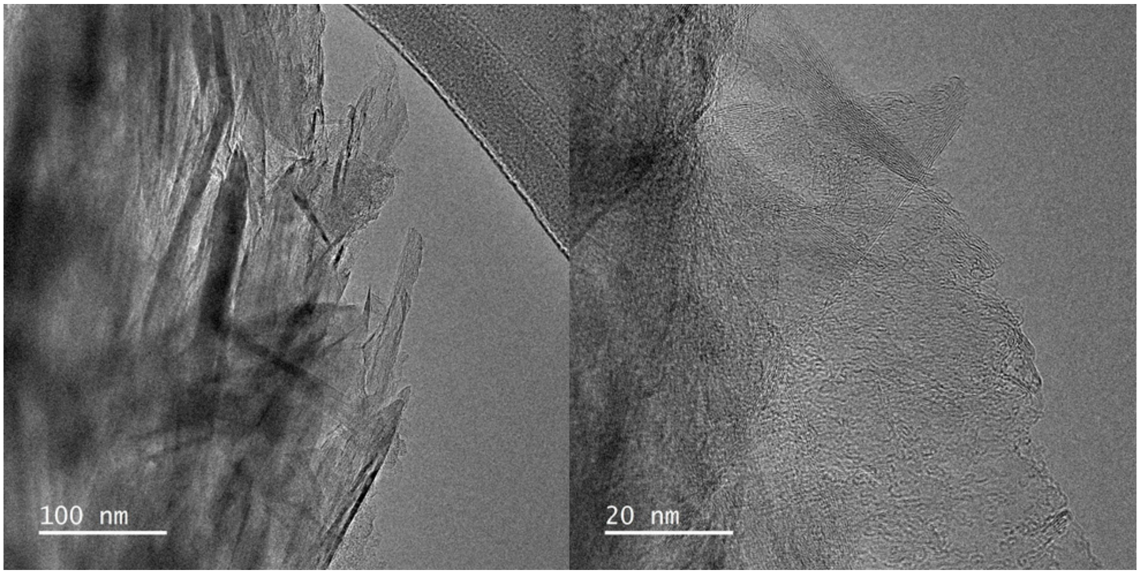

Figure S5. HR-TEM images of the CyGN.

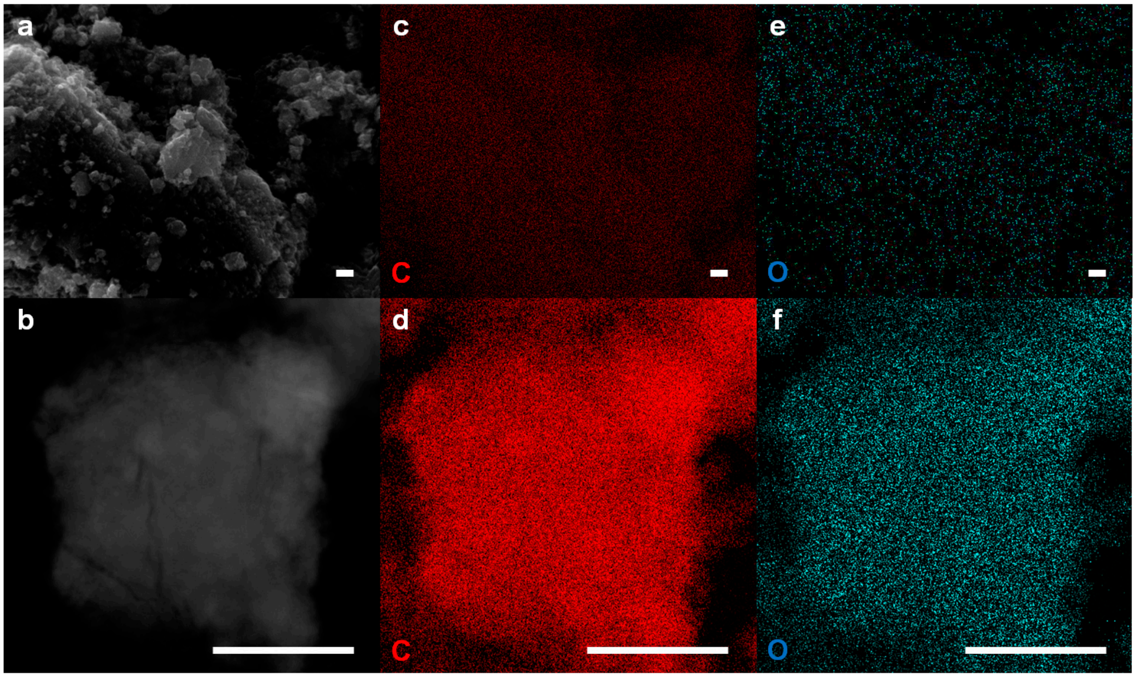

Figure S6. (a) FE-SEM and (b) HR-TEM images of the CyGN. Corresponding element mappings: (c and d) Carbon; (e and f) Oxygen. Scale bars are 1 μm.

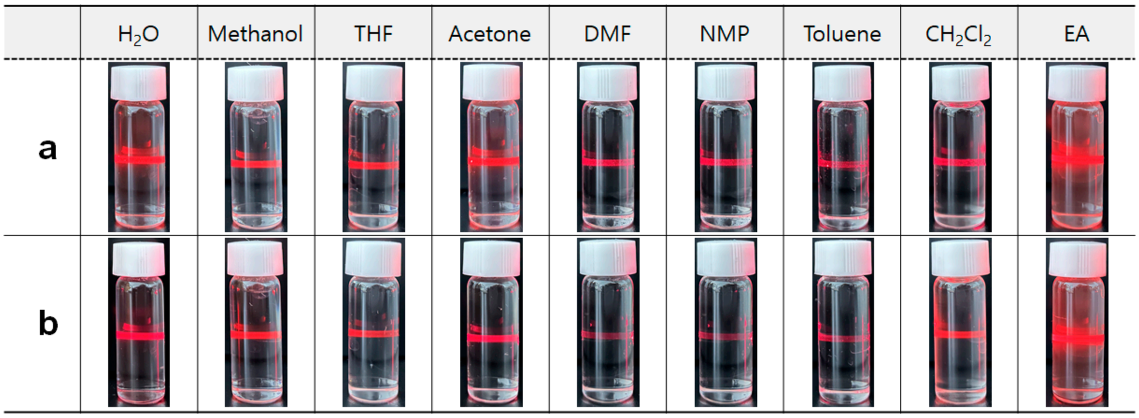

Figure S7. Photographs of the CyGN dispersed solutions in various solvents on bench top in a normal laboratory condition: (a) after 30 seconds; (b) after 1 week.

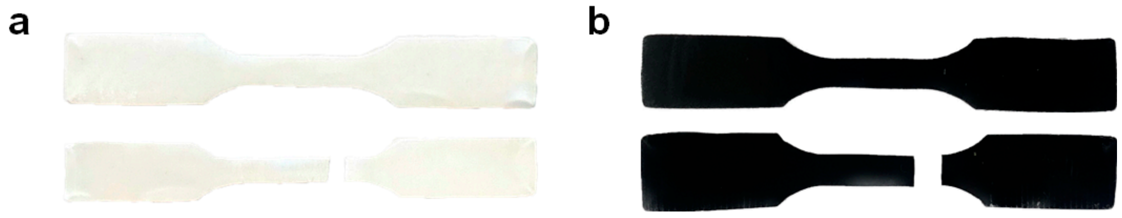

Figure S8. Photographs images of dumbbell-shaped test specimen: (a) pure ABS; (b) CyGN&ABS-1.

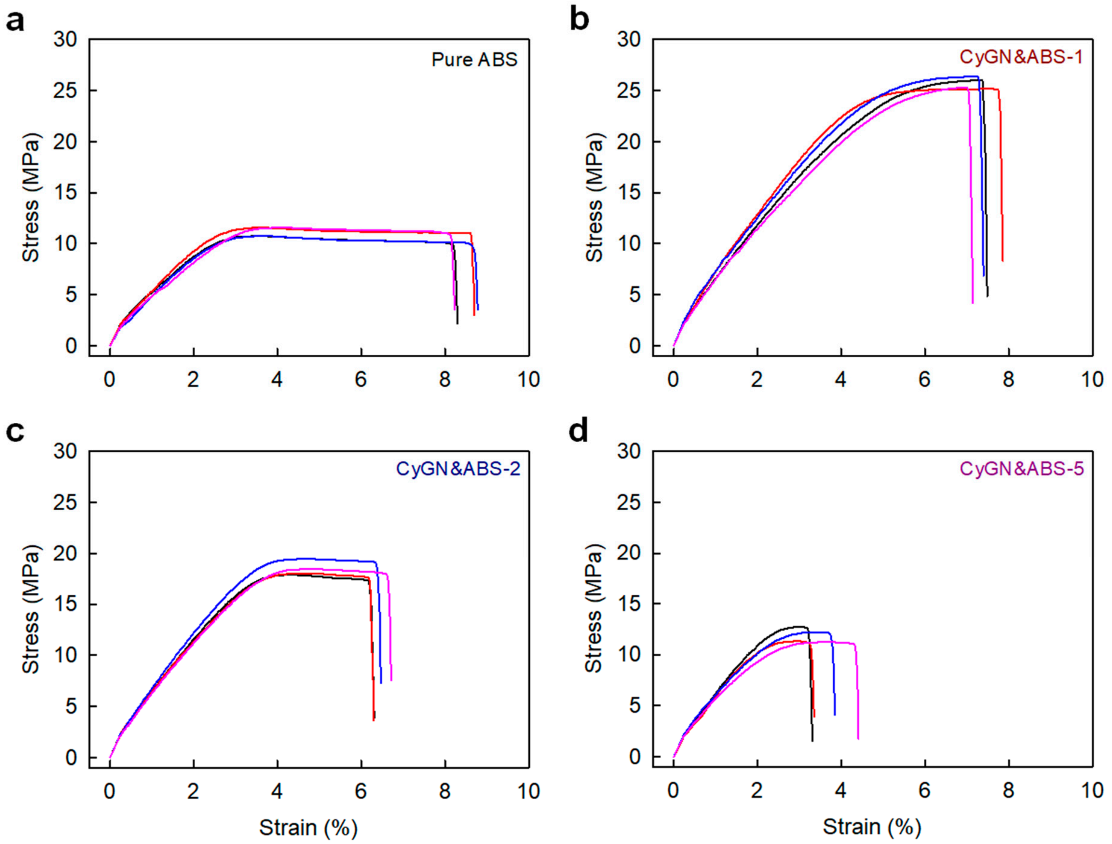

Figure S9. The stress-strain curves of the pure ABS and CyGN&ABS-X.

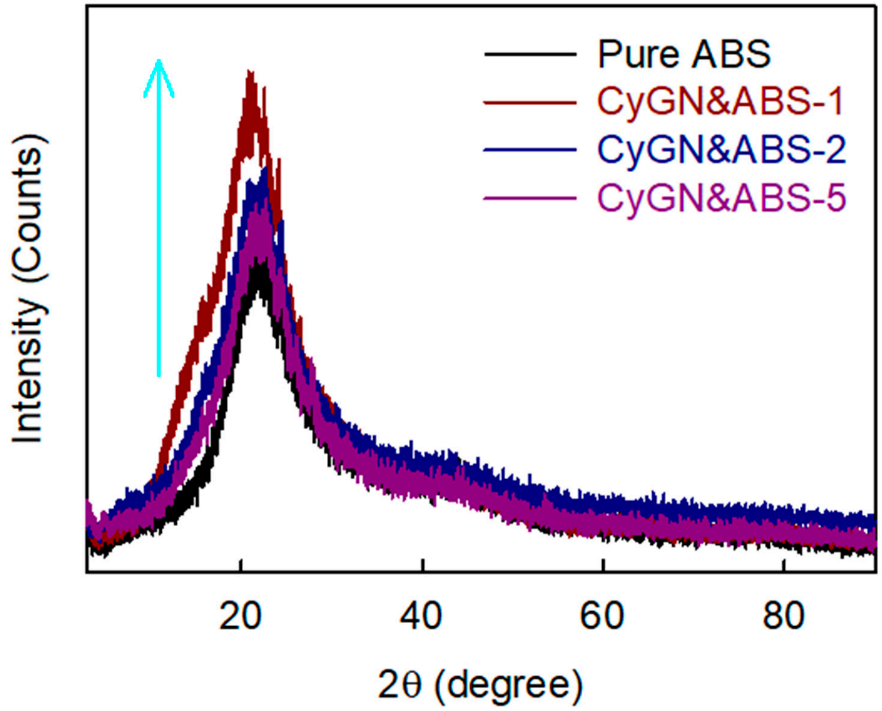

Figure S10. XRD patterns of the pure ABS and CyGN&ABS-X.

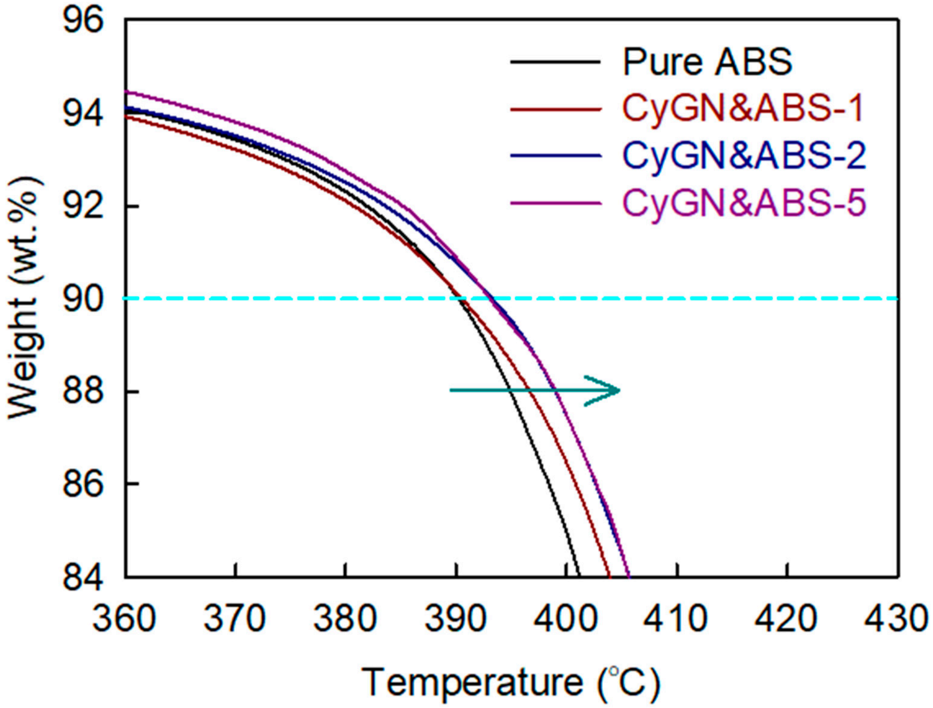

Figure S11. The magnified TGA curves of sky blue box in Figure 5a.

Table S1. TGA, EA, EDS, and XPS data of the pristine graphite and CyGN.

Sample	TGA Char Yield at 1000 °C		Element	EA (wt.%)	EDS (at.%)	XPS (at.%)
	Air	N_2				
Graphite	23.7	99.1	C	99.64	98.80	98.35
			O	0.13	1.20	1.65
CyGN	0.3	10.5	C	67.84	82.87	87.98
			O	19.14	6.70	5.73
			H	2.04	-	-
			N	6.82	10.43	6.29

Table S2. BET surface area, pore volume, and pore size of the pristine graphite and CyGN.

Sample	Surface Area (m^2/g)	Pore Volume (mL/g)	Pore Size (nm)
Graphite	2.8	0.0016	2.27
CyGN	453.4	0.4626	4.08

Table S3. Thermal properties of the pure ABS and CyGN&ABS-X.

Sample	TGA (N_2)[a]	DMA[b]
	$T_{d10\%}$ (°C)	T_g (°C)
Pure ABS	390.2	102.7
CyGN&ABS-1	390.5	104.8
CyGN&ABS-2	393.2	105.1
CyGN&ABS-5	393.1	106.6

a. The temperature at which 10% weight loss occurred on TGA thermogram obtained with heating rate of 10 °C/min. b. Glass transition temperature (T_g) determined by DMA.
